# Third-Generation Cephalosporin-Resistant Uropathogenic *Escherichia coli* From Community- and Hospital-Acquired Infections Show High Level of Antibiotic Resistance and Specific Virulence Traits

**DOI:** 10.1155/cjid/9021465

**Published:** 2025-05-04

**Authors:** Amina Bougouizi, Astri Dwyanti Tagueha, Daniela Scribano, Zohra Chekroud, Zahrat el Imen Lamraoui, Lucia Nencioni, Cecilia Ambrosi, Hamza Rahab

**Affiliations:** ^1^Research Laboratory of Interactions, Biodiversity, Ecosystems and Biotechnology, Department of Nature and Life Sciences, Faculty of Sciences, University 20 August 1955 Skikda, Skikda 21000, Algeria; ^2^Department of Public Health and Infectious Diseases, Sapienza University of Rome, Rome 00185, Italy; ^3^Biotechnology's Laboratory of the Bioactive Molecules and the Cellular Physiopathology, Department of Biology of Organisms, Faculty of Natural and Life Sciences, University of Batna2, Batna, Algeria; ^4^Department of Promotion of Human Sciences and Quality of Life, San Raffaele University, Rome, Italy; ^5^Laboratory of Microbiology of Chronic-Neurodegenerative Diseases, IRCCS San Raffaele Roma, Rome, Italy; ^6^Biotechnology Research Center - C.R.Bt Constantine, El Khroub, Algeria

**Keywords:** 3GC-resistant uropathogenic *E. coli*, Algeria, community-acquired infections, ExPEC, nosocomial infections, urinary tract infections

## Abstract

*Escherichia coli* is a leading cause of both community-acquired and nosocomial infections. In particular, *E. coli* is responsible for 90% of all uncomplicated urinary tract infections (UTIs) and 65% of complicated UTIs. Among complicated UTIs, those caused by third-generation cephalosporin (3GC)–resistant *E. coli* strains, expressing extended-spectrum beta-lactamases (ESBLs), are on the rise. These strains show often a multidrug-resistant (MDR) phenotype, limiting the therapeutic options and the increasing incidence of MDR *E. coli* in Algeria is concerning. This study aims to compare the antibiotic resistance rates and profiles as well as the virulence traits between 3CG-resistant *E. coli* isolates, collected from Algerian inpatients (IPs) and outpatients (OPs). Our analyses include phenotypic and genotypic resistance factor detection, strains classification by genotyping and phylogrouping, as well as genotypic and phenotypic virulence factor evaluation. Among 42 *E. coli* isolates, 76.20% caused UTIs. ESBL producers (*n* = 35) carried all the *bla*_CTX−M_, while *bla*_TEM_ was found in 69.04% of isolates. All isolates were MDR, and no significant differences in type and rate of antibiotic resistance were observed between IP- and OP-isolates. OP-isolates demonstrated greater virulence, exhibiting higher motility and biofilm production, compared to IP-isolates. Moreover, pathogenic Phylogroup B2 was prevalent among OP-isolates, while IP-isolates belonged predominantly to Phylogroup A. Our data suggest a uniform spreading of antibiotic-resistant genes within hospitals and communities. However, hospital environment selects for less virulent strains with increasing level of resistance; differently, communities host more virulent strains. This study highlights the urgent need to implement the surveillance of 3CG-resistant *E. coli* and to adopt the One Health approach to monitor the antimicrobial resistance (AMR) in the country.

## 1. Introduction

*Escherichia coli*, the principal facultative anaerobe found in the intestinal microbiota of humans and warm-blooded animals, exhibits a remarkable adaptability and genomic diversity [1]. While the majority of *E. coli* strains are commensal, certain strains have evolved pathogenic mechanisms capable of causing severe diseases [1, 2]. *E. coli* strains can be categorized into different phylogroups—A, B1, B2, C, D, E, and, F—indicating their animal or human origin as well as their virulent potential [3]. Indeed, pathogenic *E. coli* strains primarily belong to Phylogroups B2 and D, whereas commensal strains are predominantly found within Phylogroups A and B1 [4]. However, commensal phylogroups often display heightened resistance profiles while expressing a more limited array of virulence genes compared to pathogenic phylogroups [4, 5].

*E. coli* can be classified based on its capacity to cause infections either within the gastrointestinal tract due to intestinal pathogenic *E. coli* pathotypes (IPEC), or outside due to extraintestinal pathogenic *E. coli* strains (ExPEC) [1, 6]. The latter group can cause a variety of infections, particularly urinary tract infections (UTIs), pneumonia, bacteremia, soft tissue infections (surgical wounds), bloodstream infections, lower respiratory tract infections, and neonatal meningitis [1, 7]. Virulence genes encoding various functions crucial for adhesion, colonization, invasion, and evasion of host defenses are common features of pathogenic *E. coli* strains. These genes are often clustered in chromosomal blocks, plasmids, or phages, making transfer between different *E. coli* strains easy and contributing to the dynamic nature of their pathogenicity [1].

Uropathogenic *E. coli* (UPEC) strains are the most common pathogens responsible for approximately 90% of all uncomplicated UTIs and 65% of complicated UTIs [8]. Cystitis, a single episode of pyelonephritis, and some cases of recurrent cystitis can be classified as uncomplicated UTIs, as they are eradicated with first-line antibiotics and they result in no long-term complications. Any UTI that does not meet the above criteria or clinical management is considered a complicated UTI. Examples are UTIs in males, in immunocompromised subjects, recurrent UTIs caused by resistant strains, and hospital-acquired UTIs (HA-UTI) [9]. Risk factors of HA-UTI include catheterization, bladder dysfunction, long-term hospitalization, previous UTI history, age, and comorbidities, including diabetes, hypertension, and stroke [10]. Among HA-UTI, UPEC strains account for about 50% of total cases and the most frequently isolated from catheters [11]. Major pandemic ExPEC lineages responsible for UTIs include sequence types (ST) ST131, ST73, ST69, and ST95. However, ST131, characterized by high level of antibiotic resistance, is the predominant one in hospital settings [12]. Although there are no genomic signatures defining UPEC strains, the presence of specific urovirulence factors can support their identifications. These factors include redundant iron acquisition systems, several pili associated with specific adhesins, hemolysins, and/or cytotoxins and biofilm forming ability [13]. Accordingly, the majority of studies characterizing UPEC isolates include the analysis of urovirulence factors [13].

The management of uncomplicated UTIs is based on the use of first line antibiotic such as nitrofurantoin, sulfamethoxazole/trimethoprim, fosfomycin, and first-generation cephalosporins [14]. Differently, in complicated UTIs, the co-administration of amoxicillin plus an aminoglycoside, a second-generation cephalosporin plus an aminoglycoside, and a third-generation cephalosporin (3GC), mainly as an empirical treatment, are recommended [9].

3GC-resistant *E. coli* isolates show an increasing trend worldwide. The most critical geographical areas include African and Asian countries with percentages of 3GC-resistant isolates reaching 90% in Burkina Fasu and Bangladesh [15]. In Algeria, 3GCs are extensively prescribed for treating various infections acquired both in hospital and community settings [16, 17]. According to Algerian Antibiotic Resistance Surveillance Network (AARN) report, *E. coli* was identified as the predominant pathogen responsible for UTIs in both hospitalized and outpatient (OP) populations, accounting for 52.72% and 61.00% of isolates, respectively. Resistance to 3GC was reported in 27.84% of hospital isolates and 14.84% OP-isolates, respectively, leading to an overall resistance rate of 21.14% [18].

The widespread use of 3CGs, compounded by factors such as inadequate infection control measures and inappropriate antibiotic use, has led to a concerning rise in resistance to these agents, particularly due to the emergence of extended-spectrum β-lactamases (ESBLs) [17, 19, 20]. ESBLs are versatile enzymes responsible for the hydrolysis of beta-lactam antibiotics including penicillins and first to 3GC, and they are categorized molecularly and functionally according to Ambler and Bush Jacoby-Medeiros classifications [21, 22]*. E. coli* species mainly expresses three different ESBL types, including Cefotaximase-Munich (CTX-M), Temoniera (TEM), and sulfhydryl variable (SHV) enzymes [23]. These three ESBL types are classified in the Ambler class A and Bush Jacoby-Medeiros Group 2. In *E. coli*, the CTX-M type is the most widespread and includes 270 variants, whose genes are hosted within plasmids, transposons and integrons. They are responsible for the hydrolysis of cefotaxime, and they are found in the most resistant *E. coli* strains worldwide [23].

TEM enzymes were among the first beta-lactamases discovered, originally conferring resistance to penicillin and to the first-generation cephalosporins. Subsequently, the progressive accumulation of mutations resulted in the evolution of 202 variants with the capability to hydrolyze a broader spectrum of beta-lactams, including 3GC [24]. Moreover, TEM enzymes are also not susceptible to beta-lactamase inhibitors such as clavulanic acid and sulbactam making *E. coli* isolates resistant to amoxicillin-clavulanic acid and ampicillin-sulbactam, commonly used in community and hospitals [24]. The SHV type was initially identified in *Klebsiella pneumoniae* and showed a narrow spectrum of activity. Overtime, the 232 *E. coli* variants gained the capability to hydrolyze cephalosporins and monobactams [23]. In *E. coli*, the most represented type is CTX-M. Moreover, *bla*_CTX−M15_ gene was found to be positively associated with multidrug-resistant (MDR) *E. coli* strains. Indeed, these isolates show co-resistance to aminoglycosides and fluoroquinolones. It was suggested that the plasmidic co-carriage of plasmid-mediated quinolone resistance (PMQR) genes (i.e., *qnr* genes), and aminoglycoside methylases genes could favor the *bla*_CTX−M15_ maintenance and spreading [25]. This antibiotic-resistant gene content aligns with the alarmingly high rate of MDR *E. coli* isolates in Algeria; indeed, recent findings reported that about the 70% of clinical isolates shows an MDR phenotype. [26, 27]. Moreover, antimicrobial resistance (AMR) extends beyond hospitalized patients, affecting animals, food, the environment, and nonhospitalized populations in Algeria. Several reports highlighted the presence of about 40% of MDR *E. coli* in healthy and diseased livestock [28, 29]. The spread of ESBL-producing *E. coli* is driven by excessive antibiotic use in human and veterinary medicine [30], inadequate infection control measures, and international travels [31], which facilitates their dissemination from high-prevalence regions. Additionally, the genetic adaptability of certain lineages to the host, as ST131, causes a prolonged intestinal carriage, further expanding its transmission [32]. Carbapenems were and are the last resort antibiotics for treating ESBL-producing strains; however, their widespread use has promoted the emergence and dissemination of carbapenem-resistant strains, in several types of bacteria including *Enterobacterales* [33–35]. Although the rate of carbapenem-resistant *E. coli* strains in Algeria is still low, the identification of *bla*_OXA48_ as well as *bla*_NDM_ genes, coding for enzymes capable to hydrolyze carbapenems, is becoming a great concern [35, 36].

In light of this emerging trend, this study aims to compare the antibiotic resistance rates and profiles as well as the virulence traits between 3CG-resistant ExPEC isolates collected from Algerian inpatients (IPs) and OPs. Our analyses include phenotypic and genotypic resistance factor detection, strains classification by genotyping and phylogrouping, as well as genotypic and phenotypic virulence factor (VF) evaluation. This comprehensive investigation will increase our knowledge on 3CG-resistant *E. coli* in order to identify appropriate strategies to control their spread and to develop innovative therapeutic as well as antivirulence approaches.

## 2. Materials and Methods

### 2.1. Clinical Isolates Collection

**Figure 1:**
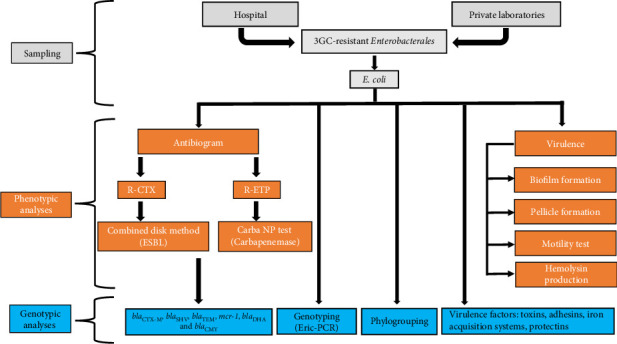
Flowchart of sample collection and genotypic and phenotypic analyses. Clinical isolates belonging to *Enterobacterales* were collected from both the public Abderrezek Bouhara Hospital and several private clinical laboratories. After testing for the resistance to 3GC, isolates belonging to *Escherichia coli* species were selected for genotypic and phenotypic analyses as described in the main text.

Clinical isolates belonging to *Enterobacterales* were collected between October 2020 and June 2022 from both the public Abderrezek Bouhara Hospital and several private clinical laboratories, selected for their large catchment area in Skikda city, Algeria. To select isolates responsible for extraintestinal infections, urine, pus, blood, and cerebrospinal fluid were screened. Isolates identified as cefotaxime-resistant based on antibiogram results provided by the clinical microbiology laboratories were included in the study. Using the formula 4PQ/L^2^ [37], the initial sample size was determined to be 29 isolates based on a 95% confidence level, 10% error margin, and 8% prevalence of *Entrobacterales* ESBL producers in Algerian clinical centers, as reported in a previous study [38]. To minimize the error margins, the sample size was expanded to 82 isolates. The work chart of sample collection and treatments is shown in Figure 1.

### 2.2. Clinical Isolates Identification and Antibiotic Susceptibility Testing

Procedures for collection, transport, and clinical isolate identification followed the clinical microbiology procedures described by Leber [39]. Clinical samples were cultured on blood agar and MacConkey agar plates (BioMérieux, France) and incubated for 24 h at 37°C. The CHROMAgar™ Orientation (DMED, Alger, Algeria) medium was also used, as previously described [35]. Isolated colonies were identified using the automated VITEK® 2 Compact 15 system (BioMérieux) and stocked in tryptic soy broth (TSB) containing 15% of glycerol at −70°C. For each experiment, isolates were freshly plated onto Lennox broth (LB) agar plates (LA, Difco, Milan, Italy) and cultured for 16 h at 37°C. VITEK 2 was also employed for antibiotic susceptibility testing. Tested antibiotics included, ampicillin (AMP), amoxicillin/clavulanic acid (AMC), piperacillin/tazobactam (TZP), cefazolin (CZ), cefoxitin (FOX), cefotaxime (CTX), ceftazidime (CAZ), imipenem (IMP), ertapenem (ETP), amikacin (AK), gentamicin (GEN), ciprofloxacin (CIP), fosfomycin (FOS), chloramphenicol (CHL), sulfamethoxazole/trimethoprim (SXT), and nitrofurantoin (NIT). The obtained minimal inhibitory concentration (MIC) values were interpreted by comparison with the standard set by Clinical Laboratory Standard Institute (CLSI 2020, Version of M02 M07 M11, 30th ed). *E. coli* strain ATCC 25922 served as the reference strain for the analysis. Additionally, the broth microdilution method was employed to ascertain the colistin MIC values following CLSI guidelines. Bacteria were classified as MDR when resistant to at least one antibiotic in three or more antibiotic categories [40].

### 2.3. Phenotypic Characterization of ESBL and Carbapenemase Production

ESBL production among isolates was evaluated through the combined disk method [41]. The production of carbapenem-hydrolyzing enzymes was assessed using the modified Carba-NP test [42].

### 2.4. Detection of Beta-Lactamase and mcr-1 Genes

DNA extraction was carried out using the boiling lysis method [35]. Briefly, isolated colonies were resuspended in 100 μL of nuclease-free water and incubated at 95°C for 10 min. After centrifugation at 10.000 × g for 10 min, supernatants were retrieved and stored at −20°C before use. DNA purity and concentration were assessed using a NanoDrop 8000 spectrophotometer (Thermo Fisher Scientific, Waltham, MA, USA). Conventional PCR was utilized to screen for the presence of the three most frequent beta-lactamase-encoding genes (*bla*_CTX−M_, *bla*_TEM_, and *bla*_SHV_), as previously described [35]. Additionally, for isolates demonstrating resistance to cefoxitin, two *ampC*-encoding genes (*bla*_DHA_ and *bla*_CMY_) were targeted, as previously described [43, 44]. Nuclease-free water was used as negative control in each reaction. Each PCR reaction was prepared according to the manufacturer's instruction, with a total volume of 15 μL, including 7.5 μL of Master Mix (Thermo Fisher Scientific), 0.2 μL of each primer pairs (forward and reverse primer solution), 1 μL of DNA template, and 6.1 μL of nuclease-free water to reach the final volume. In addition to beta-lactamase-encoding genes, the presence of *mcr*-1 gene, coding for the resistance to colistin, was screened, as previously described [45]. The primer sequences and amplicon sizes are summarized in Table 1. The PCR products were separated by electrophoresis on a 1.5% agarose gel, stained with Sybr Green, and visualized under a UV transilluminator.

### 2.5. Genotyping, Phylogrouping, and Detection of Virulence Genes

Isolates belonging to *E. coli* species were selected for genotyping and phylogrouping analyses. Isolates were genotyped using *Enterobacterial* Repetitive Intergenic Consensus sequence (ERIC)-PCR profiles as previously described [46]. Phylogenetic grouping was performed by quadruplex PCR [5, 46, 47]. Primers are reported in Table 1. Isolates not belonging to any phylogroup were excluded from further analyses. The presence of virulence genes was assessed by multiplex as well as single PCR assays as previously described [47, 48]. The banding patterns generated from ERIC-PCR were examined using TotalLab TL120 Trace Version 2006 (nonlinear dynamics) with a position tolerance set at 1.5%. Following the calculation of the Dice coefficient of similarity, cluster analysis was carried out in XLstat 7.5 (Addinsoft, USA), using the unweighted pair group method with arithmetic averages (UPGMA). To distinguish clonally distinct groups, the percentage of similarity was established at 60%.

*E. coli* isolates were tested for the presence of 24 virulence genes via a standard PCR assay. The virulence genes targeted included adhesins: *sfa/focDE* (S fimbriae or F1C fimbriae), *papC* (P fimbriae major subunit), *papG* (P fimbriae adhesin), *tsh* (temperature-sensitive hemagglutinin), *yadN* (outer membrane protein), *papAH* (P fimbriae assembly protein); toxins: *usp* (uropathogenic specific protein), *sat* (secreted autotransporter toxin), *traT* (transfer protein T), *vat* (vacuolating autotransporter toxin), *hlyA* (hemolysin A), *malX* (MalX regulator), *cnf1* (cytotoxic necrotizing factor 1), *ibeA* (invasion of brain endothelium A), *clbN* (colibactin N), *clbB* (colibactin B), siderophores: *feoB* (ferrous iron transport protein B), *iroN* (iron transport protein N), *iutA* (ferric aerobactin receptor), *fyuA* (ferric yersiniabactin receptor), *chuA* (hemin receptor), *irp2* (iron-repressible protein 2), and protectins: *kpsMT II* (capsular polysaccharide export inner-membrane protein) and *ompT* (outer membrane protease T). The UPEC reference strain CFT073 and strains B10P, I12P, and H20P, previously isolated from colonic adenoma, were used as positive controls [47, 48]. Nuclease-free water was included as negative control. Each isolate was assigned an arbitrary VF score [47].

### 2.6. Phenotypic Assays

Production of hemolysin(s) was assessed using blood agar plates (bioMérieux, Milan, Italy), whereas motility using 0.3% agar plates (Difco, Milan, Italy). Plates were incubated at 20°C for 16 h and motility zones (mm) were evaluated using ImageJ software, as previously described [49]. Biofilm formation was measured as previously reported [47]. Briefly, overnight cultures were diluted 1:10 in fresh LB (Difco, Milan, Italy) and used to inoculate three replicate wells per microtiter plate (200 μL per well). Negative controls consisted of uninoculated wells containing sterile medium only and the *E. coli* strain D4C was used as positive control [47, 50]. After reading the absorbance at 600 nm (OD_600_), plates were washed three times with phosphate-buffer saline (PBS) solution, fixed with methanol for 20 min at room temperature, and stained with 0.1% crystal violet solution (Sigma, SIAL, Italy) for 15 min. After four additional washes with water, the surface-associated dye was solubilized with 200 μL of 33% acetic acid and the OD_570_ was recorded. Results were reported as the OD_570_/OD_600_ ratio to normalize the amount of biofilm formed to the total bacterial content. The pellicle formation was assayed as previously described [50]. Ten microliters of overnight cultures were transferred into test tubes containing 3 mL of fresh LB. Tubes were incubated for 24 h at 37°C in static condition. Pellicles were photographed with a Xiaomi MI 10T phone. Sedimentation assay was performed as previously described [51]. Briefly, overnight cultures were diluted 1:100 in fresh LB (total volume 5 mL) and incubated statically at 37°C for 24 h. One hundred microliter samples were taken from the middle of the cultures, and the OD_600_ was measured using a 1-cm cuvette in a total volume of 1 mL. The OD_600_ value of 0.8 was chosen as a cut-off to distinguish nonaggregative and aggregative cells. Representative images were acquired with a Xiaomi MI 10T phone.

### 2.7. Statistical Analysis

The nonparametric data category was assigned to both the antibiotic effectiveness and the presence of virulence genes. Therefore, Wilcoxon matched-pairs signed rank test was used to determine the different distribution of these two variables among OP- and IP-isolates. Regarding the impact of virulence genes in multiple antibiotic susceptibility/resistance patterns, the binary logistic regression analysis was conducted to evaluate an association. The coefficient regression represents the likelihood (odds ratio = OR); therefore, VF genes with significant OR were recognized as predictors of susceptibility or resistance to antibiotic agents. Additionally, a permutational multivariate analysis of variance (PERMANOVA) test with 9999 permutations was performed to evaluate the differences in phenotypic characteristics among phylogroups and origins, followed by a sequential Bonferroni post hoc test. *p* values less than 0.05 were considered statistically significant. Nonparametric and logistic regression analyses were performed using IBM SPSS Statistics, Version 23.0 (IBM Corporation, Cary, NC, USA), and PERMANOVA test was executed using Past software Version 4.11 (Oslo, Norway).

## 3. Results

### 3.1. 3GC-Resistant *E. coli* Isolates Collected From OPs and IPs Belong to Different Phylogroups

A total of 82 isolates belonging to *Enterobacterales* and resistant to 3GC were collected, with 37 from IPs and 45 from OPs. The identification results showed that the highest proportion of isolates were *E. coli* (*n* = 47; 57.32%), followed by *K*. *pneumoniae* (34.00%). Four isolates (4.90%), including *Enterobacter cloacae* and *Proteus mirabilis*, *Serratia marcescens*, and *Salmonella* spp., were individually found in distinct specimens.

**Figure 2:**
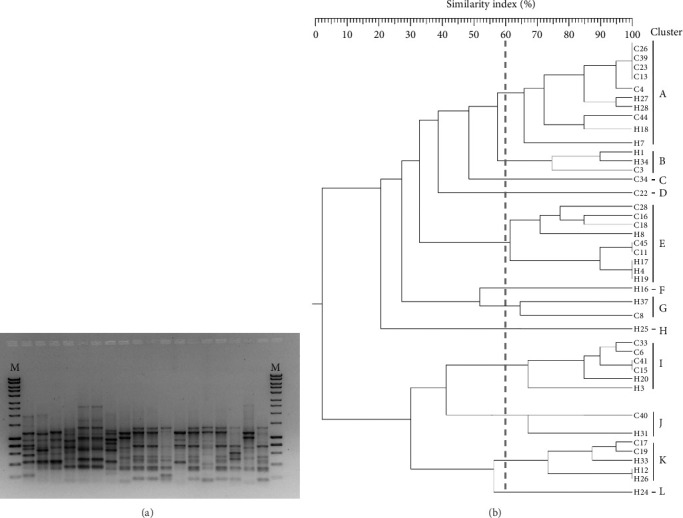
ERIC-PCR profiles and dendrogram of IP- and OP-isolates. Representative image of ERIC-PCR profiles on agarose gel. (a) Genetic similarity among isolates was based on the Dice Similarity Index as the numeric coefficient and the unweighted pair group mathematical average (UPGMA) clustering algorithm. Similarity percentage cutoff of 60% was set to differentiate cluster profiles (b) M: 1 Kb DNA marker.

Focusing on *E. coli*, we found 22 isolates collected from urine specimens, representing 100% of *E. coli* collected from patients admitted to private laboratories (OP-isolates, *n* = 22); differently, we found 10 isolates from urine, nine from pus and one from blood specimens, representing the 50%, 45%, and 5% of isolates collected from hospitalized patients (IP-isolates, *n* = 20), respectively. ERIC-PCR genotyping generated bands ranging in size from about 100 bp to 3000 bp, with the majority ranging from about 400 bp to 1000 bp (Figure 2(a)). The analysis of the dendrogram (60% of genetic similarity) showed 7 clusters comprising more than 1 isolate (Figure 2(b)) and 5 singletons (C34, C22, H16, H25, H24), indicating isolates with unique profiles. IP- and OP-*E. coli* were homogenously distributed within the identified clusters. The analysis of phylogroup distribution showed a high prevalence of B2 isolates among OP-isolates (9 out of 22; 41%), followed by Phylogroup E (6 out of 22; 27.30%), while, IP-isolates belonged mainly to Phylogroup A (10 out of 20; 50.00%), followed by B2 (6 isolates), as reported in Table 2.

### 3.2. OP- and IP-*E. coli* Isolates Share Similar Antibiotic Resistance Profiles

OP- and IP-isolates showed similar profiles of antibiotic resistance, with no statistically significant differences. All isolates were resistant to ampicillin, cefazolin, and cefotaxime (MIC ≥ 32 and 64 respectively). Moreover, 68.18% of OPs and 90.00% of IPs showed co-resistance to ceftazidime (MIC ≥ 32). IPs exhibited higher rates of resistance to amoxicillin/clavulanic acid (70.00% vs. 59.10%, MIC ≥ 32), piperacillin/tazobactam (50.00% vs. 36.40%, MIC ≥ 64), and ciprofloxacin (90.00% vs. 72.73%, MIC ≥ 4). In addition, a higher percentage of IP-isolates showed increased MIC values compared to OP-isolates for ceftazidime (44.44% vs. 33.33%, MIC ≥ 64). Conversely, isolates from OPs showed higher resistance rates to gentamicin (45.50% vs. 30.00%, MIC ≥ 16) and chloramphenicol (27.27% vs. 5.00%, MIC ≥ 64). Additionally, the resistance rates to trimethoprim/sulfamethoxazole were high in both groups, but higher in OP-isolates (90.90% vs. 80.00%, MIC ≥ 320). Both groups had similar resistance rates to nitrofurantoin and fosfomycin (5.00% of IPs vs. 4.60% of OPs, MIC ≥ 128 and MIC ≥ 256, respectively) as well as to amikacin (30.00% of IPs vs. 36.40% of OPs, MIC ≥ 16). Carbapenems remained effective against all OP-isolates but one, whereas 25.00% and 15.00% of the IP-isolates showed nonsusceptibility to ertapenem and imipenem, respectively. The detailed MIC results are provided in Table S1.

OP- and IP-isolates were all MDR. In particular, 19.00% of isolates were resistant to one agent in three different antibiotic categories, while the 81.00% showed resistance to one agent in more than four different categories (Table S2). OP-isolates belonging to Phylogroup B2 accounted for the highest resistance rates against the majority of tested antibiotics, while, among IPs, isolates from Phylogroup A showed the greater resistance rates (Table S2).

### 3.3. *bla*_CTX−M_ Is the Dominant β-lactamase Gene Type Among IP- and OP-Isolates

Among the 3GC-resistant *E. coli* isolates, 35 (83.30%) were identified as ESBL producers, including 15 isolates from IPs and 20 isolates from OPs. Additionally, 6 *E. coli* were found to be carbapenemase producers, including 5 from IPs and one from OPs. The *bla*_CTX−M_ gene was the most prevalent, being found in all, but two, ESBL-producing isolates, followed by the *bla*_TEM_ gene, found in 69.04% of the isolates. The less represented genes were *bla*_CMY_, *bla*_DHA_, and *bla*_SHV_ found in 4, 2, and 2 isolates, respectively (Table S1).

### 3.4. Specific Virulence Genes Characterize OP and IP Isolates

It is known that ExPEC isolates possess a high carriage of virulence genes [1]. Results showed a significant difference of virulence gene content between OP- and IP-isolates (*p* = 0.0025). Moreover, OP- and IP-isolates belonging to the Phylogroup B2 showed the highest VF score compared to isolates belonging to other phylogroups (B2 vs. other phylogroups of OP-isolates, *p* < 0.001; B2 vs. other phylogroups of IP-isolates, *p* < 0.001). The most prevalent genes in both groups were those encoding for iron acquisition systems, such as *fyuA*, detected in 38 isolates (90.50%), followed by *feoB* in 35 isolates (83.30%) and *irp2* in 33 isolates (78.60%); conversely, the least represented was *iroN* (4.60% of OP-isolates and 15.00% of IP-isolates, respectively). *fyuA* was significantly associated with IP-isolates (100% from IPs vs. 81.80% from OP, *p* = 0.045) (Table 3); vice versa *chuA* gene was associated with OP-isolates (86.40% from OPs vs. 40.00% from IPs, *p* = 0.002). Among the analyzed toxin-encoding genes, *usp*, *sat*, *malX*, and *cnf1* genes were more frequently found in OP-isolates compared to IP-isolates, with the most notable difference for the *cnf1* gene (22.70% vs. 5.00%), followed by *malX* (36.40% vs. 25.00%), *sat* (40.90% vs. 30.00%), and *usp* (36.40% vs. 30.00%) (Table 3). However, these differences were not statistically significant (*p* > 0.05). In contrast, *traT* gene was more prevalent among hospital isolates (80.00% of IPs vs. 63.60% of OPs). The least frequent genes found in both groups were *vat* (4.60% from OPs vs. 5.00% from IPs), *hlyA* (4.60% vs. 0.00%), *clbB* (4.60% vs. 5.00%), and *clbN* (9.10% vs. 5.00%). The *ibeA* virulence gene was absent in both *E. coli* groups.

**Figure 3:**
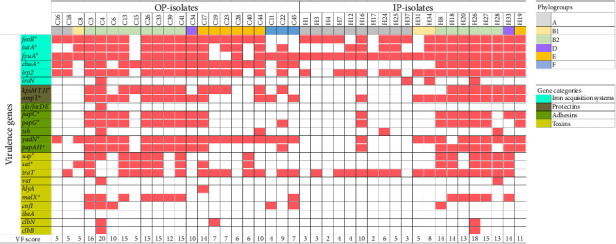
Distribution of virulence genes among *Escherichia coli* isolates from outpatients (OP-isolates) and inpatients (IP-isolates). The presence/absence of genes is indicated by filled and empty boxes, respectively. Phylogroups and gene categories are indicated by colored boxes. Asterisks underline genes significantly associated with resistance/susceptibility to ertapenem, gentamicin, and ciprofloxacin antibiotics. The virulence factor (VF) score is also reported.

Genes encoding fimbriae-associated factors, such as *papC*, *papG*, and *papAH,* were more frequently detected in OP-isolates, although this association did not reach statistical significance (*p* > 0.05). Interestingly, the presence of the *yadN* gene was significantly higher in OP- compared to IP-isolates (90.90% vs. 50.00%, *p* = 0.003) (Table 3). Differently, *tsh* and *sfa* genes were barely detected in both *E. coli* groups. The protectin-encoding genes, *kpsMT* II (59.10% vs. 35.00%) and *ompT* (59.10% vs. 55.00%), were both more common in *E. coli* from OPs than from IPs. However, the differences in prevalence for both genes were not statistically significant (*p* > 0.05) (Table 3). The prevalence of virulence genes and the relative VF scores for each isolate are reported in Figure 3.

### 3.5. Specific Association Between Virulence Genes and Resistance Phenotype to Commonly Used Antibiotics

According to the binary logistic regression analysis, significant associations were identified between certain virulence genes and resistance or susceptibility to three antibiotics: ertapenem, ciprofloxacin, and gentamicin (Table 4). The *pap* genes (*papC* and *papG*: OR = 0.500, *p* = 0.022; *papAH*: OR = 0.472, *p* = 0.016), along with *sat* (OR = 0.583, *p* = 0.049) and *chuA* (OR = 0.077, *p* = 0.009), are associated with susceptibility to ertapenem, with no VFs showing an inverse association (Table 4). In contrast, resistance to gentamicin is predicted by several virulence genes, including *pap* genes and *ompT* that showed the strongest association (OR > 10, *p* < 0.001), followed by *yadN* (OR = 9.625, *p* = 0.019), *malX* (OR = 8.625, *p* = 0.002), *chuA* (OR = 6.036, *p* = 0.024), *kpsMTII* (OR = 5.500, *p* = 0.013), *sat* (OR = 5.250, *p* = 0.014), *usp* (OR = 4, *p* = 0.040), and *iutA* (OR = 2.481, *p* = 0.001) (Table 4). Additionally, resistance to ciprofloxacin is also associated with several virulence genes, including the two *pap* genes (*papC*: OR = 2.125, *p* = 0.006; *papAH*: OR = 7.875, *p* = 0.039), *malX* (OR = 1.619, *p* = 0.035), *fyuA* (OR = 19.800, *p* = 0.003), and *ompT* (OR = 14.636, *p* = 0.005). The only VF significantly associated with gentamicin sensitivity is *feoB* (OR = 0.741, *p* = 0.031). The predictor role of selected virulence genes for resistance to gentamicin and ciprofloxacin was confirmed by the MIC values.

### 3.6. Isolates Collected From OPs Show High Biofilm-Forming Activity and Motility

**Figure 4:**
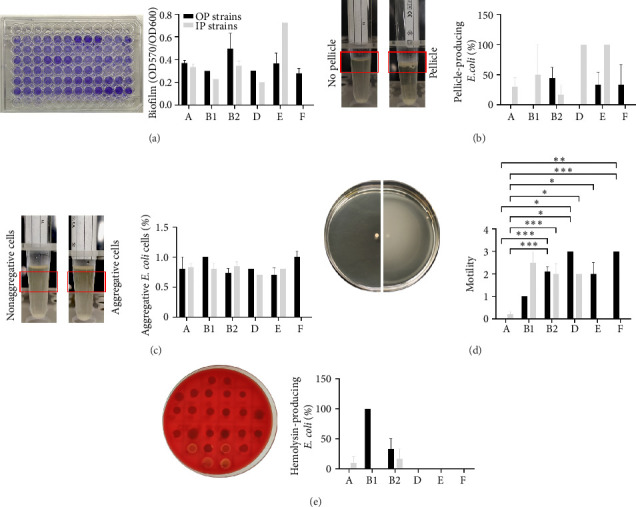
Phenotypic traits of IP- and OP-*Escherichia coli* isolates. (a) Representative image of the crystal violet stained biofilm on a 96-well tissue culture plate (left panel) and relative distribution of isolates according to the phylogroups (right panel). Isolates were grouped based on A_570_/A_600_ values: no production (0 < OD < 0.2), weak production (0.2 < OD < 0.4), moderate production (0.4 < OD < 0.6), and strong production (OD > 0.6). (b) Representative images of non- and pellicle-forming isolates (left panel) and relative distribution of isolates according to the phylogroups (right panel). (c) Representative images of nonaggregative and aggregative *E. coli* isolates (left panel) and relative distribution of isolates according to the phylogroups (right panel). (d) Representative images of *E. coli* motility on a soft agar plate (left panel) and relative distribution of isolates according to the phylogroups (right panel). Isolates were grouped based on the diameter size of swim zones: 0, nonmotile (< 10 mm); 1, low motile (10–20 mm); 2, motile (20–40 mm), and 3, hypermotile (> 40 mm). (e) Representative image of hemolysin-producing *E. coli* isolates on blood agar plates (left panel) and relative distribution of isolates according to the phylogroups (right panel) and the source (right). Black and gray bars indicate OP-isolates and IP-isolates, respectively.

Biofilm represents one of the common strategies used by *E. coli* to establish infections; therefore, the biofilm-forming ability of each isolate was measured. Results demonstrated that OP-isolates produced higher amounts of biofilm than IP-isolates which showed mainly weak biofilm-forming activity (*p* < 0.0001), and this phenotype was not phylogroup-dependent (Figure 4(a)). The capability to form air–liquid interface pellicle was also evaluated. Results showed that the 33.40% of isolates produced pellicle; however, no significant difference in the distribution of pellicle-positive or -negative isolates between OP- and IP-isolates was found (Figure 4(b)). Interestingly, the majority of pellicle-positive isolates were weak biofilm producers (8/14, 57.00%). Furthermore, the sedimentation assay showed that the 69.00% of isolates showed OD_600_ values higher than 0.8, which was the selected cut-off to distinguish aggregative and non-aggregative cells, independently from the isolation source (Figure 4(c)). Nonaggregative phenotype was mainly associated to isolates showing weak biofilm forming activity (22/29).

In *E. coli,* motility is considered a VF, especially associated to ExPEC strains. Hence, the extent of motility for each isolate was tested. Results showed that OP-isolates were more motile compared to IP-isolates (*p* < 0.018). This phenotype was mainly associated to B2 (*p* = 0.0004) and D (*p* = 0.0463) (Figure 4(d)). Isolates belonging to Phylogroup A were almost not motile, whereas OP-isolates of Phylogroup F were highly motile (*p* = 0.0035).

### 3.7. OP- and IP-*E. coli* Isolates Show Low Hemolytic Activity

Commonly, ExPEC isolates are characterized by the presence of toxins [1]. Each isolate was tested for the production of hemolysins on blood agar plates. Few isolates showed hemolysis (*n* = 6; 14.30%), with 4 (18.20%) from OPs and 2 (10.00%) from IPs. Among hemolytic isolates, 50.00% belonged to Phylogroup B2, while the remainder belong to Phylogroups A and B1 (Figure 4(e)). However, none of the six hemolytic isolates possessed the *hlyA* gene, suggesting the involvement of other cytolytic toxins.

## 4. Discussion

3GC-resistant *Enterobacterales* are categorized in the critical group of the WHO bacterial priority pathogens list [52]. In this two-year study, a total of 82 3GC-resistant *Enterobacterales* isolates from IPs and OPs were collected in Skikda, Algeria. Among these, *E. coli* predominated, accounting for 57.10% of the isolates. This finding aligns with numerous studies highlighting *E. coli* as the most prevalent species in both hospital- and community-acquired enterobacterial infections [53, 54]. Among these, 76.20% of isolates were recovered from urine samples of both IPs and OPs, confirming their major involvement in both community- and hospital-acquired UTIs [55, 56]. An in-depth phenotypic and genotypic characterization of 3GC-resistant *E. coli* collected from IPs and OPs was performed.

Unlike many studies reporting higher resistance rates among isolates from IPs compared to those from OPs [57, 58], our study found that isolates from IPs and OPs exhibited nonstatistically significant differences in resistance rates to the majority of tested antibiotics. This finding corroborates results from Milano et al. and suggests comparable selective pressures from antibiotic use in both hospital and community settings [55].

The majority of 3GC-resistant *E. coli* isolates were identified as ESBL producers, consistent with findings from North Africa and other regions [16]. In Algeria, studies have reported a high prevalence of 3GC resistance, predominantly linked to *bla*_CTX−M_ genes [29, 59]. Similar studies in Tunisia and Morocco have also identified *bla*_CTX−M−15_ as the predominant variant, often coexisting with *bla*_TEM_ and *bla*_SHV_ [60, 61]. In accordance, our study showed that all ESBL-producing *E. coli,* but two, carried the *bla*_CTX−M_ gene, confirming its primary involvement in cefotaxime inactivation. Moreover, a significant percentage harbored also *bla*_TEM_, while only two isolates showed the *bla*_SHV_ indicating that the distribution of this latter gene is still limited, as previously observed [60, 61]. Interestingly, 35.00% of isolates retained the susceptibility to the cefoxitin, a non-ESBL susceptible antibiotic; however, the *ampC*-type encoding genes *bla*_CMY_ and *bla*_DHA_ were detected in 26.66% and 13.33% of cefoxitin-resistant *E. coli*, respectively. This result suggests that *bla*_CMY_ and *bla*_DHA_ could be involved in cefoxitin inactivation and encourages the monitoring of the spread of these genes. We observed a higher prevalence of carbapenem-resistant isolates among IP-isolates compared to OP-isolates. Although this difference did not reach a statistical significance, probably due to the low number of identified isolates, this result is in line with previous observations on the low rate of carbapenem-resistant strains collected from OPs [35, 62, 63]. The limited occurrence outside hospital settings is likely due to the reserved use of carbapenems as last-line antibiotics for treating severe infections in hospitals [62]. Among, ertapenem-resistant isolates, the *NDM-5* gene was found, highlighting its predominant role in carbapenem resistance in *E. coli* isolates [62–64]. However, variations in carbapenemase distribution have been reported across different regions, influenced by differences in antimicrobial prescribing practices, infection control measures, and surveillance strategies. In Algeria, most studies have focused on hospital settings, where OXA-48- and NDM-5-producing *E. coli* have been predominantly reported [36]. However, sporadic cases in community-acquired infections have also been documented [36, 62] suggesting the possible circulation of these resistant strains outside hospitals. Studies from North Africa and neighboring regions have documented the presence of carbapenemase-producing *E. coli* in both hospital and community settings, with some reports indicating an increasing detection of these resistance determinants outside healthcare environments [65–67]. The problem of carbapenem-resistant *Enterobacteriaceae* (CRE) in Africa is aggravated by factors such as high infection rates, poor diagnostic tools, sub-optimal disease surveillance, and misuse of antibiotics [67].

Our findings highlighted high resistance levels to fluoroquinolones and sulfamethoxazole/trimethoprim in both groups of isolates, in agreement with previous reports showing resistance rates exceeding 50% to these agents among resistance to 3GC enterobacteria in Tunisia [60, 64]. Similar patterns were observed in 3GC-resistant *E. coli* isolates from both IPs and OPs in Ushuaia, *Argentina*, with resistance rates up to 75% for ciprofloxacin and 65% for sulfamethoxazole/trimethoprim [56, 60]. This observation may be attributed to the easy availability and widespread, uncontrolled use of these antibiotics in these countries, as in Algeria, and strengthens the correlation between resistance to 3GC and resistance to other antibiotic classes due to shared resistance mechanisms or cross-resistance.

The susceptibility of both groups to amikacin and fosfomycin, along with low resistance to chloramphenicol and nitrofurantoin, is supported by previous reports [68, 69]. These antibiotics may be effective alternatives for treating 3GC-resistant infections in both hospital and community settings. The observed susceptibility of all isolates to colistin may be due to its limited use in human therapy in Algeria, where it is more commonly used in veterinary medicine for growth promotion or the prevention and treatment of *Enterobacterales* infections in animals [70]. These findings are consistent with the fact that resistance to colistin among clinical *Enterobacterales* in humans has been documented in only few studies in Algeria [38, 71–73].

Results from genotyping revealed a genetic relatedness among IP- and OP-isolates, suggesting cross-contaminations between hospital and community settings. Accordingly, only 12.00% of isolates showed unique genotypic profiles. This could justify the similar resistance rates and profiles found in both groups of isolates. The expansion of successful clusters combined with the intrinsic genetic variability of *E. coli* underscores the complexity of dissemination dynamics and genetic evolution of this species, aligning with the observations of Saeki et al. [74]. Accordingly, a low level of genotypic differences was observed among *E. coli* sharing the same profile of antibiotic resistance and, in particular, among ESBL-producing *E. coli* [75]. Differently, we found a significant difference in the distribution of phylogroups among IP and OP-*E. coli* isolates; IP-*E. coli* mainly belonged to Phylogroups A and B1, whereas the pathogenic Phylogroups B2 and E were more prevalent in OP-isolates. This distribution has been consistently reported in other studies [76, 77]. Indeed, it is interesting to note that carbapenem-resistant isolates found in this study belonged to Phylogroups A and B1, further supporting the hypothesis that commensal *E. coli* strains, upon acquiring high levels of antibiotic resistance, are primarily responsible for nosocomial infections. On the other hand, the high prevalence of the pathogenic Phylogroups B2 and E in OPs suggests that community settings may favor the persistence of more virulent strains. Indeed, we found that OP-*E. coli* isolates exhibited a significant higher virulence gene content compared to IP-isolates. Among the genes tested, *yadN* and *chuA* were statistically significantly associated with OP-isolates, while *fyuA* was mainly present in IP-isolates. *yadN* encodes for the major subunit of the Yad fimbriae expressed by UPEC during bladder–cell adhesion and biofilm formation [78, 79]. Accordingly, OP-isolates showed a higher biofilm-forming activity compared to IP-isolates. *chuA* mediates direct heme uptake, instead *fyuA* is the receptor for yersiniabactin uptake [79, 80]. Although functional redundancy exists in the iron acquisition systems of *E. coli*, *chuA* and *fyuA* are two receptors that contribute the most during infections and in particular during UTIs. The *chuA* gene showed higher prevalence in B2 and E isolates in comparison to A and B1 isolates [81]. Moreover, the prevalence of *chuA* among OP-*E. coli* was already reported in previous studies [27, 53, 81]. This indicates that the heme receptor better copes with the limiting-iron conditions experienced by community-associated *E. coli*. This finding highlights the adaptability of *E. coli*, and suggests that the heme receptor is crucial for bacterial survival and pathogenicity under iron-limiting conditions [82]. Conversely, the higher prevalence of *fyuA* in isolates from hospitalized patients was already reported [83, 84]. Moreover, a significant association between this gene and the resistance to ceftazidime and cefotaxime was also noted [83, 84] confirming the contribution of commensal *E. coli* to nosocomial infections. It can be concluded that bacteria preferentially retain the most efficient iron acquisition systems in their genome to adapt to diverse environmental conditions. Although iron acquisition is not directly involved in antibiotic resistance, it enhances bacterial pathogenicity by providing a competitive edge, enabling them to survive and persist under antibiotic exposure and immune challenges.

The binary regression analysis identified a positive association between several virulence genes and the resistance to antibiotics commonly used in therapy. Specifically, the presence of *pap* genes, *malX*, and *ompT* strongly correlates with resistance to ciprofloxacin and gentamicin. This aligns with findings from previous studies reporting similar associations [85, 86]. Yazdanpour et al. noted a significant association between *malX* and increased resistance to ciprofloxacin and gentamicin, as well as 3GC [86], which supports our results. *malX* is located in a UPEC pathogenetic island and its role is still not well characterized [87]. Recently, it has been shown that this gene correlates with UTIs and the phylogenetic group B2, which includes mainly ExPEC such as UPEC strains [88]. Furthermore, it was suggested that this gene could increase *E. coli* fitness in urine, being involved in the uptake of different carbohydrates [88]. Hence, it can be hypothesized that antibiotic-resistant UPEC strains harboring *malX* may be more proficient in urinary tract colonization, driven by an enhanced capacity for nutrient uptake within the bladder environment. Furthermore, Monroy-Pérez et al. observed that *ompT* and *pap* genes were frequently present in strains resistant to ciprofloxacin and gentamicin [89]. *pap* genes significantly contribute to UTIs, by aiding resistant UPEC strains to adhere to the urothelium. Moreover, it has been recently reported that OmpT and OmpT-like proteases, strongly associated to UPEC strains, could degrade antimicrobial peptides in the urinary tract, thereby conferring resistance to innate immunity [90]. Overall, it can be speculated that the energetic cost of resistance mechanisms may be compensated by virulence genes, collectively enhancing the fitness and persistence of UPEC strains. However, a study conducted by Adegoke et al. on cefotaxime-resistant *E. coli* isolated from a wastewater treatment plant showed a correlation between *pap* genes, *malX*, and *ompT* with resistance to ciprofloxacin and gentamicin [91]. The potential combined carriage of these genes in numerous isolates raises the possibility that specific virulence and antibiotic-resistant genes may co-localize on similar genetic elements, such as plasmids or integrons [92].

The phenotypic screening showed that OP-isolates were biofilm-formers and motile, while IP-isolates were characterized by weak biofilm activity and lower level of motility. In *E. coli,* biofilm represents a relevant VF of UPEC strains since it helps the establishment of UTIs [93]. Furthermore, it was shown that biofilm-forming activity is a feature that outlines *E. coli* strains undergoing pathogenic adaptation [47]. Hence, in line with several reports, community-associated *E. coli* isolates must enhance the expression of specific virulence traits to cause infections, such as biofilm-forming activity and motility [27, 53]. Accordingly, successful hospital-associated *E. coli* are primarily forced at acquiring antibiotic resistance mechanisms, rather than virulence traits, thereby retaining the typical features of commensal *E. coli* [94].

The dissemination of 3GC-resistant *E. coli* in both hospital and community settings underscores the need for reinforced surveillance programs and optimized antibiotic therapy in Algeria. Infection control strategies should be adapted to limit the spread of resistant strains, particularly in OPs where we found more virulent isolates. Therefore, strengthening antimicrobial stewardship programs by extending preventive measures beyond healthcare facilities is essential to mitigate the public health impact of these infections. With this aim, the WHO recently developed the “Tricycle” protocol within the One Health approach, to highlight the concept of simultaneously addressing three aspects of bacterial resistance, human health, food chain (animals), and the environment. The unique bioindicator, or sentinel, analyzed in the tricycle protocol is represented by ESBL-producing *E. coli* strains due to (i) their variable colonization rates in and among countries, and prevalence trends in humans, in farm animals, and in the environment; (ii) interventions leading to a decreased exposure to antibiotics in animals or humans have been followed by a decrease in ESBL *E. coli* occurrence rates; (iii) ESBLs confer resistance to critically important antimicrobial drugs, including the current rise of carbapenem as well as colistin-resistant ESBL-producing *E. coli* strains. Hence, ESBL *E. coli* is a relevant and representative proxy for the magnitude and trends of the global AMR problem. Based on the tricycle protocol, data on ESBL-producing *E. coli* strains offer the opportunity to explore the prevalence and to track the dissemination of AMR [95].

This study presents some limitations. The sample size was relatively small, and isolates were collected from a single region, which may not fully represent the national epidemiological situation. Additionally, whole-genome sequencing was not performed, which could have provided a more comprehensive understanding of the genetic background of resistance and VFs as well as the dissemination of variants. Future studies should expand the sample size and use advanced molecular techniques to further elucidate the mechanisms driving resistance and pathogenicity in 3GC-resistant *E. coli*.

## 5. Conclusion

Results described here reported a high level and homogeneous distribution of resistance rates among 3CG-resistant *E. coli* isolated from community- and hospital-acquired infections in Algeria. Interestingly, IP- and OP-isolates belonged to different phylogroups and were characterized by the presence of specific virulence genes and by the expression of particular virulence traits. It can be hypothesized that the success of OP-isolates is mainly associated with their capability to form biofilm and to express motility, while IP-isolates rely mainly on the expression of antibiotic-resistant genes. This study highlights the urgent need to implement the surveillance of 3CG-resistant *E. coli* and to adopt the One Health approach to monitor AMR in the country. Ultimately, integrating genomic and phenotypic data is essential to develop innovative therapeutic solutions, new antibacterial molecules, phage-based therapies, and antivirulence strategies, to combat these MDR strains and to optimize AMR control policies.

## Figures and Tables

**Table 1:** List of primer sequences used in the PCR reactions for detection of resistance genes and for phylogenetic grouping.

Primer function	Target	Primer sequence	Amplicon size (bp)	Reference
*beta-lactamase* genes amplification	*bla*_CTX−M_	F-TTTGCGATGTGCAGTACCAGTAA	500	[35]
		R-CGATATCGTTGGTGGTGCCATA		
	*bla*_TEM_	F-ATGAGTATTCAACAT TTC CG	840	[35]
		R-CCAATGCTTAATCAG TGA GG		
	*bla*_SHV_	F-TTTATGGCGTTACCTTTGACC	1051	[35]
		R-ATTTGTCGCTTCTTTACTCGC		
	*bla*_CMY_	F-ATGATGAAAAAATCGTTATGC	1200	[43]
		R-TTGCAGCTTTTCAAGAATGCGC		
	*bla*_DHA_	F-TGATGGCACAGCAGGATATTC	997	[44]
		R-GCTTTGACTCTTTCGGTATTCG		

*Colistin-resistant gene*	*mrc-1*	F-AGTCCGTTTGTTCTTGTGGC	320	[45]
		R-AGATCCTTGGTCTCGGCTTG		

Phylotyping	*chuA*	F-ATGGTACCGGACGAACCAAC	288	[5]
		R- TGCCGCCAGTACCAAAGACA		
	*yjaA*	F- CAAACGTGAAGTGTCAGGAG	211	[5]
		R-AATGCGTTCCTCAACCTGTG		
	*TspE4*.C2	F-CACTATTCGTAAGGTCATCC	152	[5]
		R-AGTTTATCGCTGCGGGTCGC		
	*arpA*	F-AACGCTATTCGCCAGCTTGC	400	[5]
		R-TCTCCCCATACCGTACGCTA		
	Group E	F-GATTCCATCTTGTCAAAATATGCC	301	[5]
		R-GAAAAGAAAAAGAATTCCCAAGAG		
	Group C	F-AGTTTTATGCCCAGTGCGAG	219	[5]
		R-TCTGCGCCGGTCACGCCC		
	Internal control	F-CGGCGATAAAGACATCTTCAC	489	[5]
		R-GCAACGCGGCCTGGCGGAAG		

**Table 2:** Distribution of phylogroups among IP- and OP-*Escherichia coli* isolates.

Isolates	Phylogroups *n* (%)					
	B2 (*n* = 15)	D (*n* = 2)	A (*n* = 12)	B1 (*n* = 3)	E (*n* = 7)	F (*n* = 3)
IPs-*E. coli* (*n* = 20)	6 (30%)	1 (5%)	10 (50%)	2 (10%)	1 (5%)	0 (0%)
OPs-*E. coli* (*n* = 22)	9 (40.9%)	1 (4.6%)	2 (9.1%)	1 (4.6%)	6 (27.3%)	3 (13.6%)

**Table 3:** Prevalence of virulence genes (VGs) in IP- and OP-*Escherichia coli* isolates.

Gene categories	VGs	OP-*E. coli* (*n* = 22), *n* (%)	IP-*E. coli* (*n* = 20), *n* (%)	*p* value
Toxins	*usp*	8 (36.4)	6 (30)	0.662
	*sat*	9 (40.9)	6 (30)	0.461
	*traT*	14 (63.6)	16 (80)	0.241
	*vat*	1 (4.6)	1 (5)	0.945
	*malX*	8 (36.4)	5 (25)	0.426
	*hlyA*	1 (4.6)	0 (0)	0.335
	*cnf1*	5 (22.7)	1 (5)	0.101
	*ibeA*	0 (0)	0 (0)	—
	*clbN*	2 (9.1)	1 (5)	0.607
	*clbB*	1 (4.6)	1 (5)	0.945

Adhesins	*sfa/focDE*	1 (4.6)	0 (0)	0.335
	*papC*	10 (45.5)	8 (40)	0.721
	*papG*	11 (50)	7 (35)	0.327
	*tsh*	2 (9.1)	2 (10)	0.920
	*yadN*	20 (90.9)	10 (50)	**0.003**
	*papAH*	11 (50)	9 (45)	0.516

Iron acquisition systems	*feoB*	18 (81.8)	17 (85)	0.782
	*ironN*	1 (4.6)	3 (15)	0.249
	*iutA*	15 (68.2)	13 (65)	0.827
	*fyuA*	18 (81.8)	20 (100)	**0.045**
	*chuA*	19 (86.4)	8 (40)	**0.002**
	*irp2*	17 (77.3)	16 (80)	0.830

Protectins	*kpsMT II*	13 (59.1)	7 (35)	0.118
	*ompT*	13 (59.1)	11 (55)	0.789

*Note:* Numbers in bold indicate statistically significant *p* values.

**Table 4:** Binary logistic regression analysis of virulence-associated genes as predictors of resistance to antibiotics.

VGs	Resistance to antibiotic agents					
	Ertapenem		Gentamicin		Ciprofloxacin	
	OR	*p*	OR	*p*	OR	*p*
*usp*	—	> 0.05	**4**	**0.040**	—	> 0.05
*sat*	**0.583**	**0.049**	**5.250**	**0.014**	—	> 0.05
*malX*	—	> 0.05	**8.625**	**0.002**	**1.619**	**0.035**
*papC*	**0.500**	**0.022**	**80.500**	**<** **0.001**	**2.125**	**0.006**
*papG*	**0.500**	**0.022**	**80.500**	**<** **0.001**	-	> 0.05
*yadN*	—	> 0.05	**9.625**	**0.019**	—	> 0.05
*papAH*	**0.472**	**0.016**	**61.600**	**<** **0.001**	**7.875**	**0.039**
*feoB*	—	> 0.05	**0.741**	**0.031**	—	> 0.05
*iutA*	—	> 0.05	**2.481**	**0.001**	-	> 0.05
*fyuA*	—	> 0.05	—	> 0.05	**19.800**	**0.003**
*chuA*	**0.077**	**0.009**	**6.036**	**0.024**	—	> 0.05
*kpsMT II*	—	> 0.05	**5.500**	**0.013**	—	> 0.05
*ompT*	—	> 0.05	**23.800**	**<** **0.001**	**14.636**	**0.005**

*Note:* A significant association (*p* ≤ 0.05) was identified by the binary logistic regression analysis. When *p* ≤ 0.05 and OR > 1, the virulence-associated gene is a predictor of resistance to the antibiotic agent. However, when OR < 1, the virulence-associated gene is a predictor of susceptibility to the antibiotic agent. Numbers in bold indicate statistically significant values.

## Data Availability

The data of this study are available from the corresponding authors upon reasonable request.

## Supporting Information

Table S1: Distribution of MIC values and β-lactamase encoding genes among IP- and OP-*E. coli* isolates.

Table S2: Prevalence and phylogenetic distribution of antibiotic resistant IP- and OP- *E. coli* isolates.
