# Supplementary material for: Third-Generation Cephalosporin-Resistant Uropathogenic Escherichia coli From Community- and Hospital-Acquired Infections Show High Level of Antibiotic Resistance and Specific Virulence Traits
Source: Can J Infect Dis Med Microbiol. 2025 May 4;2025:9021465. doi: 10.1155/cjid/9021465 (PMC12066185; doi:10.1155/cjid/9021465)
Supplement: Supporting Information — Additional supporting information can be found online in the Supporting Information section. [file 9021465.f1.docx]

**Supplementary materials**

**Table S1:** Antibiotic resistance and the distribution of genes encoding β-lactamase rate of IP- and OP*-E. coli* strains

| **SAMPLE ID** | **MIC values (µg/ml)** | | | | | | | | | | | | | | | | **β-lactamase encoding genes** |
| --- | --- | --- | --- | --- | --- | --- | --- | --- | --- | --- | --- | --- | --- | --- | --- | --- | --- |
|  | **AMP** | **AMC** | **TZP** | **CZ** | **FOX** | **CTX** | **CAZ** | **ETP** | **IMP** | **AK** | **GEN** | **CIP** | **FOS** | **NIT** | **CHL** | **SXT** |  |
| C3 | ≥32 (R) | ≥32 (R) | 64  (R) | ≥64 (R) | 16 | ≥64 (R) | 32  (R) | 0,25 | ≤0,25 | 16  (R) | ≥16 (R) | ≥4 (R) | ≤16 | 32 | 16 | ≥320 (R) | *blaCTX-M* |
| C4 | ≥32 (R) | 16 | 64  (R) | ≥64 (R) | 32 (R) | ≥64 (R) | ≥64 (R) | 0,25 | ≤0,25 | ≤2 | ≥16 (R) | ≥4 (R) | ≤16 | ≤16 | ≥64 (R) | ≥320 (R) | *blaCTX-M- blaTEM* |
| C6 | ≥32 (R) | 16 | 8 | ≥64 (R) | 16 | ≥64 (R) | 32  (R) | ≤ 0,12 | ≤0,25 | 16  (R) | ≥16 (R) | ≥4 (R) | ≤16 | 32 | 16 | ≥320 (R) | *blaCTX-M* |
| C8 | ≥32 (R) | 4 | ≤4 | ≥64 (R) | ≤4 | ≥64 (R) | 8 | ≤ 0,12 | ≤0,25 | ≤2 | ≥16 (R) | ≥4 (R) | ≤16 | ≤16 | ≥64 (R) | ≥320 (R) | *blaCTX-M- blaTEM* |
| C11 | ≥32 (R) | 4 | ≤4 | ≥64 (R) | 16 | ≥64 (R) | 8 | ≤ 0,12 | ≤0,25 | ≤2 | ≤1 | ≥4 (R) | ≤16 | ≤16 | ≥64 (R) | ≤20 | *blaCTX-M* |
| C13 | ≥32 (R) | ≥32 (R) | 64  (R) | ≥64 (R) | ≥64 (R) | ≥64 (R) | ≥64 (R) | ≤ 0,12 | ≤0,25 | 16  (R) | ≥16 (R) | ≥4 (R) | ≤16 | 64 | 16 | ≥320 (R) | *blaCTX-M* |
| C15 | ≥32 (R) | ≥32 (R) | ≥128 (R) | ≥64 (R) | ≤4 | ≥64 (R) | 1 | ≤ 0,12 | ≤0,25 | 16  (R) | ≤1 | ≥4 (R) | ≤16 | ≤16 | 4 | ≥320 (R) | *blaCTX-M- blaTEM* |
| C16 | ≥32 (R) | ≥32 (R) | ≥128 (R) | ≥64 (R) | 8 | ≥64 (R) | 8 | ≤ 0,12 | ≤0,25 | ≤2 | ≤1 | ≤0,25 | ≤16 | 32 | 8 | ≥320 (R) | *blaCTX-M- blaTEM* |
| C17 | ≥32 (R) | 16 | 64  (R) | ≥64 (R) | 32 (R) | ≥64 (R) | ≥64 (R) | 0,25 | ≤0,25 | 8 | ≥16 (R) | ≥4 (R) | ≤16 | ≤16 | 16 | ≥320 (R) | *blaCTX-M- blaTEM* |
| C18 | ≥32 (R) | ≥32 (R) | ≥128 (R) | ≥64 (R) | ≤4 | ≥64 (R) | 32  (R) | 0,25 | 2 | ≤2 | ≤1 | ≥4 (R) | ≤16 | ≤16 | 16 | ≤20 | *blaCTX-M* |
| C19 | ≥32 (R) | ≥32 (R) | ≥128 (R) | ≥64 (R) | 8 | ≥64 (R) | 32  (R) | ≤ 0,12 | ≤0,25 | ≤2 | ≤1 | ≤0,25 | ≤16 | 64 | 8 | ≥320 (R) | *blaCTX-M- blaTEM* |
| C22 | ≥32 (R) | ≥32 (R) | ≤4 | ≥64 (R) | ≥64 (R) | ≥64 (R) | 32  (R) | ≤ 0,12 | ≤0,25 | ≤2 | ≤1 | ≤0,25 | ≤16 | ≤16 | 8 | ≥320 (R) | *blaCTX-M* |
| C23 | ≥32 (R) | 4 | ≤4 | ≥64 (R) | 8 | ≥64 (R) | 8 | ≤ 0,12 | ≤0,25 | ≤2 | ≤1 | ≤0,25 | ≤16 | 64 | 4 | ≥320 (R) | *blaCTX-M* |
| C26 | ≥32 (R) | ≥32 (R) | 8 | ≥64 (R) | ≤4 | ≥64 (R) | 32  (R) | ≤ 0,12 | ≤0,25 | 16  (R) | ≥16 (R) | ≥4 (R) | ≤16 | ≤16 | ≤2 | ≥320 (R) | *blaCTX-M- blaTEM* |
| C28 | ≥32 (R) | 16 | ≤4 | ≥64 (R) | 32 (R) | ≥64 (R) | 32  (R) | ≤ 0,12 | ≤0,25 | ≤2 | ≤1 | ≥4 (R) | ≤16 | 32 | ≥64 (R) | ≥320 (R) | *blaCTX-M- blaTEM* |
| C33 | ≥32 (R) | 16 | 8 | ≥64 (R) | ≤4 | ≥64 (R) | 32  (R) | ≤ 0,12 | ≤0,25 | 8 | ≤1 | ≥4 (R) | ≤16 | ≤16 | 4 | ≥320 (R) | *blaCTX-M* |
| C34 | ≥32 (R) | ≥32 (R) | ≥128 (R) | ≥64 (R) | ≤4 | ≥64 (R) | ≥64 (R) | ≤ 0,12 | ≤0,25 | 16  (R) | ≥16 (R) | ≥4 (R) | ≤16 | ≤16 | 4 | ≥320 (R) | *blaCTX-M- blaTEM* |
| C39 | ≥32 (R) | ≥32 (R) | 64  (R) | ≥64 (R) | ≥64 (R) | ≥64 (R) | 32  (R) | ≤ 0,12 | ≤0,25 | 16  (R) | ≥16 (R) | ≥4 (R) | ≤16 | ≤16 | 4 | ≥320 (R) | *blaCTX-M- blaTEM- blaDHA* |
| C40 | ≥32 (R) | 8 | ≤4 | ≥64 (R) | ≤4 | ≥64 (R) | 8 | ≤ 0,12 | ≤0,25 | ≤2 | ≤1 | ≤0,25 | ≤16 | ≤16 | ≥64 (R) | ≥320 (R) | *blaCTX-M- blaTEM* |
| C41 | ≥32 (R) | ≥32 (R) | ≥128 (R) | ≥64 (R) | ≥64 (R) | ≥64 (R) | ≥64 (R) | ≤ 0,12 | 2 | 16  (R) | ≥16 (R) | ≥4 (R) | ≤16 | >128 (R) | 8 | ≥320 (R) | *blaCTX-M- blaTEM* |
| C44 | ≥32 (R) | ≥32 (R) | ≥128 (R) | ≥64 (R) | ≤4 | ≥64 (R) | 4 | 2 (R) | 2 | ≤2 | ≤1 | ≤0,25 | ≤16 | ≤16 | 4 | ≥320 (R) | *blaTEM- blaOXA-48* |
| C45 | ≥32 (R) | ≥32 (R) | ≥128 (R) | ≥64 (R) | ≥64 (R) | ≥64 (R) | 32  (R) | 0,25 | ≤0,25 | ≤2 | ≤1 | ≥4 (R) | ≥256 (R) | ≤16 | ≥64 (R) | ≥320 (R) | *blaTEM-blaSHV- blaCMY* |
| H1 | ≥32 (R) | 16 | ≤4 | ≥64 (R) | ≤4 | ≥64 (R) | 32  (R) | ≤ 0,12 | ≤0,25 | ≤2 | ≤1 | ≤0,25 | ≤16 | ≤16 | 16 | ≥320 (R) | *blaCTX-M-blaTEM-blaSHV* |
| H3 | ≥32 (R) | ≥32 (R) | ≥128 (R) | ≥64 (R) | ≥64 (R) | ≥64 (R) | ≥64 (R) | ≥8 (R) | 2 | ≤2 | ≤1 | ≥4 (R) | ≤16 | ≤16 | 16 | ≥320 (R) | *blaTEM-blaCMY- blaNDM-5* |
| H4 | ≥32 (R) | ≥32 (R) | ≥128 (R) | ≥64 (R) | ≥64 (R) | ≥64 (R) | ≥64 (R) | ≥8 (R) | ≥16 (R) | ≤2 | ≤1 | ≥4 (R) | ≤16 | ≤16 | 16 | ≥320 (R) | *blaTEM-blaCMY- blaNDM-5* |
| H7 | ≥32 (R) | 4 | ≤4 | ≥64 (R) | 16 | ≥64 (R) | 32  (R) | ≤ 0,12 | ≤0,25 | ≤2 | ≤1 | ≤0,25 | ≤16 | >128 (R) | 4 | ≤20 | *blaCTX-M* |
| H8 | ≥32 (R) | 16 | 32 | ≥64 (R) | 16 | ≥64 (R) | ≥64 (R) | ≤ 0,12 | ≤0,25 | 16  (R) | ≥16 (R) | ≥4 (R) | ≤16 | 64 | 16 | ≥320 (R) | *blaCTX-M* |
| H12 | ≥32 (R) | ≥32 (R) | ≥128 (R) | ≥64 (R) | ≤4 | ≥64 (R) | 32  (R) | ≤ 0,12 | ≤0,25 | ≤2 | ≤1 | ≥4 (R) | ≤16 | ≤16 | 16 | ≥320 (R) | *blaCTX-M- blaTEM* |
| H16 | ≥32 (R) | ≥32 (R) | ≥128 (R) | ≥64 (R) | 8 | ≥64 (R) | 32  (R) | 0,25 | 1 | ≤2 | ≤1 | ≥4 (R) | ≤16 | ≤16 | 16 | ≤20 | *blaCTX-M* |
| H17 | ≥32 (R) | ≥32 (R) | ≥128 (R) | ≥64 (R) | ≥64 (R) | ≥64 (R) | ≥64 (R) | ≥8 (R) | 2 | ≤2 | ≤1 | ≥4 (R) | ≤16 | ≤16 | 16 | ≥320 (R) | *blaTEM-blaCMY- blaNDM-5* |
| H18 | ≥32 (R) | ≥32 (R) | 16 | ≥64 (R) | 16 | ≥64 (R) | ≥64 (R) | ≤ 0,12 | ≤0,25 | 16  (R) | ≥16 (R) | ≥4 (R) | ≥256 (R) | 32 | 16 | ≥320 (R) | *blaCTX-M- blaTEM* |
| H19 | ≥32 (R) | ≥32 (R) | 64  (R) | ≥64 (R) | ≤4 | ≥64 (R) | 32 (R) | ≤ 0,12 | ≤0,25 | 16  (R) | ≥16 (R) | ≥4 (R) | ≤16 | ≤16 | 4 | ≥320 (R) | *blaCTX-M- blaTEM* |
| H20 | ≥32 (R) | 16 | 8 | ≥64 (R) | ≤4 | ≥64 (R) | 32 (R) | ≤ 0,12 | ≤0,25 | 8 | ≥16 (R) | ≥4 (R) | ≤16 | ≤16 | 4 | ≥320 (R) | *blaCTX-M- blaTEM* |
| H24 | ≥32 (R) | ≥32 (R) | 64  (R) | ≥64 (R) | ≥64 (R) | ≥64 (R) | ≥64 (R) | ≤ 0,12 | ≤0,25 | 16  (R) | ≤1 | ≥4 (R) | ≤16 | ≤16 | 16 | ≥320 (R) | *blaCTX-M- blaTEM* |
| H25 | ≥32 (R) | ≥32 (R) | ≥128 (R) | ≥64 (R) | ≤4 | ≥64 (R) | 32  (R) | ≤ 0,12 | ≤0,25 | ≤2 | ≤1 | ≥4 (R) | ≤16 | ≤16 | 16 | ≥320 (R) | *blaCTX-M- blaTEM* |
| H26 | ≥32 (R) | ≥32 (R) | ≥128 (R) | ≥64 (R) | ≤4 | ≥64 (R) | 32  (R) | ≤ 0,12 | ≤0,25 | 16  (R) | ≥16 (R) | ≥4 (R) | ≤16 | ≤16 | 8 | ≥320 (R) | *blaCTX-M* |
| H27 | ≥32 (R) | ≥32 (R) | ≥128 (R) | ≥64 (R) | ≤4 | ≥64 (R) | 32  (R) | ≤ 0,12 | ≤0,25 | 8 | ≥16 (R) | ≥4 (R) | ≤16 | ≤16 | 4 | ≥320 (R) | *blaCTX-M- blaTEM* |
| H28 | ≥32 (R) | 8 | ≤4 | ≥64 (R) | ≤4 | ≥64 (R) | 4 | ≤ 0,12 | ≤0,25 | 4 | ≤1 | ≥4 (R) | ≤16 | ≤16 | 4 | ≥320 (R) | *blaCTX-M- blaTEM* |
| H31 | ≥32 (R) | ≥32 (R) | ≥128 (R) | ≥64 (R) | ≥64 (R) | ≥64 (R) | ≥64 (R) | ≥8 (R) | ≥16 (R) | 16  (R) | ≤1 | ≥4 (R) | ≤16 | ≤16 | 4 | ≥320 (R) | *blaCTX-M- blaTEM- blaNDM-5* |
| H33 | ≥32 (R) | ≥32 (R) | ≤4 | ≥64 (R) | 32 (R) | ≥64 (R) | 32  (R) | ≤ 0,12 | ≤0,25 | ≤2 | ≤1 | ≥4 (R) | ≤16 | ≤16 | 16 | ≤20 | *blaCTX-M- blaDHA* |
| H34 | ≥32 (R) | ≥32 (R) | ≥128 (R) | ≥64 (R) | ≥64 (R) | ≥64 (R) | ≥64 (R) | 2 (R) | ≥16 (R) | 8 | ≤1 | ≥4 (R) | ≤16 | ≤16 | 4 | ≤20 | *blaCTX-M- blaTEM- blaNDM-5* |
| H37 | ≥32 (R) | 4 | ≤4 | ≥64 (R) | ≤4 | ≥64 (R) | 1 | ≤ 0,12 | ≤0,25 | ≤2 | ≤1 | ≥4 (R) | ≤16 | ≤16 | ≥64 (R) | ≥320 (R) | *blaCTX-M- blaTEM* |

AMP, ampicillin; AMC, amoxicillin/clavulanic acid; TZP, piperacillin/tazobactam; CZ, cefazolin; FOX, cefoxitin; CTX, cefotaxime; CAZ, ceftazidime; IMP, imipenem; ETP, ertapenem; AK, amikacin; GEN, gentamicin; CIP, ciprofloxacin; FOS, fosfomycin; CHL, chloramphenicol; SXT, sulfamethoxazole/trimethoprim; NIT, nitrofurantoin.

**Table S2:** Prevalence and phylogenetic distribution of antibiotic-resistant IP- and OP- *E. coli* strains

| Antibiotics | *OP-E. coli*  (n=22) | | | | | | *IP-E. coli*  (n=20) | | | | |
| --- | --- | --- | --- | --- | --- | --- | --- | --- | --- | --- | --- |
|  | **B2 (n=9); n(%)** | **A (n=2); n(%)** | **B1**  **(n=1); n(%)** | **D (n=1); n(%)** | **E (n=6);**  **n(%)** | **F (n=3); n(%)** | **B2 (n=6); n(%)** | **A (n=10); n(%)** | **B1 (n=2); n(%)** | **D (n=1); n(%)** | **E (n=1); n(%)** |
| AMP | 9 (100%) | 2 (100%) | 1 (100%) | 1 (100%) | 6 (100%) | 3 (100%) | 6 (100%) | 10 (100%) | 2 (100%) | 1 (100%) | 1 (100%) |
| AMC | 6 (66,67%) | 2 (100%) | 0 (0%) | 1 (100%) | 2 (33,33%) | 2 (66,67%) | 3 (50%) | 7 (70%) | 2 (100%) | 1 (100%) | 1 (100%) |
| TZP | 2 (22,22%) | 2 (100%) | 0 (0%) | 1 (100%) | 2 (33,33%) | 1 (33,33%) | 2 (33,33%) | 6 (60%) | 2 (100%) | 0 (0%) | 0 (0%) |
| CZ | 9 (100%) | 2 (100%) | 1 (100%) | 1 (100%) | 6 (100%) | 3 (100%) | 6 (100%) | 10 (100%) | 2 (100%) | 1 (100%) | 1 (100%) |
| FOX | 4 (44,44%) | 0 (0%) | 0 (0%) | 0 (0%) | 2 (33,33%) | 2 (66,67%) | 0 (0%) | 4 (40%) | 2 (100%) | 1 (100%) | 0 (0%) |
| CTX | 9 (100%) | 2 (100%) | 1 (100%) | 1 (100%) | 6 (100%) | 3 (100%) | 6 (100%) | 10 (100%) | 2 (100%) | 1 (100%) | 1 (100%) |
| CAZ | 8 (88,89%) | 1 (50%) | 0 (0%) | 1 (100%) | 3 (50%) | 2 (66,67%) | 5 (88,33%) | 9 (90%) | 2 (100%) | 1 (100%) | 1 (100%) |
| ETP | 0 (0%) | 0 (0%) | 0 (0%) | 0 (0%) | 1 (16,67%) | 0 (0%) | 0 (0%) | 3 (30%) | 2 (100%) | 0 (0%) | 0 (0%) |
| IMP | 0 (0%) | 0 (0%) | 0 (0%) | 0 (0%) | 0 (0%) | 0 (0%) | 0 (0%) | 1 (10%) | 2 (100%) | 0 (0%) | 0 (0%) |
| AK | 0 (0%) | 0 (0%) | 0 (0%) | 0 (0%) | 0 (0%) | 0 (0%) | 0 (0%) | 0 (0%) | 0 (0%) | 0 (0%) | 0 (0%) |
| GEN | 7 (77,78%) | 0 (0%) | 1 (100%) | 1 (100%) | 1 (16,67%) | 0 (0%) | 5 (83,33%) | 0 (0%) | 0 (0%) | 0 (0%) | 1 (100%) |
| CIP | 9 (100%) | 1 (50%) | 1 (100%) | 1 (100%) | 2 (33,33%) | 2 (66,67%) | 6 (100%) | 8 (80%) | 2 (100%) | 1 (100%) | 1 (100%) |
| FOS | 1 (11,11%) | 0 (0%) | 0 (0%) | 0 (0%) | 0 (0%) | 0 (0%) | 0 (0%) | 0 (0%) | 1 (50%) | 0 (0%) | 0 (0%) |
| NIT | 1 (11,11%) | 0 (0%) | 0 (0%) | 0 (0%) | 0 (0%) | 0 (0%) | 0 (0%) | 1 (10%) | 0 (0%) | 0 (0%) | 0 (0%) |
| CHL | 0 (0%) | 1 (50%) | 1 (100%) | 0 (0%) | 2 (33,33%) | 2 (66,67%) | 0 (0%) | 1 (10%) | 0 (0%) | 0 (0%) | 0 (0%) |
| SXT | 9 (100%) | 1 (50%) | 1 (100%) | 1 (100%) | 6 (100%) | 2 (66,67%) | 6 (100%) | 8 (80%) | 1 (100%) | 0 (0%) | 1 (100%) |

*Color code indicates antibiotic categories: Penicillins, Cephalosporins, Carbapenems, Aminoglycosides, Fluoroquinolones, Fosfomycin, Nitrofurantoin, Chloramphenicol, Trimethroprim-sulfamethoxazole.
